# Hepatic fatty acid biosynthesis in KK‐A^y^ mice is modulated by administration of persimmon peel extract: A DNA microarray study

**DOI:** 10.1002/fsn3.728

**Published:** 2018-07-20

**Authors:** Ryoichi Izuchi, Tomoko Ishijima, Shinji Okada, Keiko Abe, Yuji Nakai

**Affiliations:** ^1^ Toyo Institute of Food Technology Kawanishi Japan; ^2^ Graduate School of Agricultural and Life Sciences The University of Tokyo Bunkyo‐ku Japan; ^3^ Project on Health and Anti‐aging Kanagawa Academy of Science and Technology Life Science & Environment Research Center Kawasaki Japan

**Keywords:** DNA microarray, fatty acid biosynthesis, ketone body, obese diabetic KK‐A^y^ mouse, persimmon peel

## Abstract

**Scope:**

Previously, we showed that the intake of a persimmon peel (PP) extract altered hepatic gene expression associated with the insulin signaling pathway and enhanced tyrosine phosphorylation of insulin receptors in nonobese type 2 diabetic Goto‐Kakizaki rats. Our objective was to evaluate the effect of fat‐soluble PP extract on obese type 2 diabetic KK‐A^y^ mice with insulin resistance.

**Methods and results:**

KK‐A^y^ mice were fed a diet mixed with 0.1% of the extract for 8 weeks. The total ketone body levels in the plasma of PP extract‐fed mice were significantly lower than those in the normal diet‐fed mice. Hepatic nonesterified palmitic acid content was higher in the PP extract‐fed mice than in normal diet‐fed mice. The hepatic gene expression profiles of the treated mice indicated upregulation of fatty acid synthesis and downregulation of inflammation‐associated genes, predicting SREBP‐1c and PPARγ activation.

**Conclusion:**

These results suggest that the PP extract enhances hepatic fatty acid synthesis via SREBP‐1c and PPARγ, as well as anti‐inflammatory activity in KK‐A^y^ mice.

## 1 INTRODUCTION

The incidence of type 2 diabetes mellitus induced by obesity is increasing; therefore, certain approaches are required to prevent or reduce its deterioration. Adipocyte hypertrophy and visceral fat accumulation resulting from excessive energy intake cause insulin resistance, which can lead to obesity‐induced diabetes (Schuster, 2010). In patients with diabetes mellitus, the promotion of glucose uptake into cells by insulin and consequent activation of fatty acid synthesis are essential for the reduction of blood glucose. Therefore, control of lipid metabolism is essential for the prevention and improvement of type 2 diabetes mellitus.

Persimmon, *Diospyros kaki* L., is a fruit tree cultivated primarily in East Asian countries. The compounds present in persimmon fruit are expected to protect our body against diabetes mellitus by reducing insulin resistance: β‐cryptoxanthin, aproanthocyanidin and vitamins C and E (Endemann, Goetz, Edmond, & Brunengraber, 1982; Fujiwara, Yoshioka, Yoshioka, Ushiyama, & Horikoshi, 1988; Gorinstein et al., 2001; Hosack, Dennis, Sherman, Lane, & Lempicki, 2003). High amounts of carotenoids, polyphenols, and vitamins (Gorinstein et al., 2001) are present in the persimmon peel (PP); however, mostly, the peel is wasted because its benefits have not been thoroughly explored.

For the effective use of PP, we prepared a fat‐soluble PP extract (Izuchi, Takahashi, & Inada, 2009) and administered it to Goto‐Kakizaki (GK) rats, a nonobese type 2 diabetes model (Izuchi et al., 2011). Our previous study showed that GK rats fed a PP extract showed altered hepatic gene expression associated with the insulin signaling pathway. Moreover, hepatic tyrosine phosphorylation of the insulin receptor beta‐subunit was enhanced by the intake of PP extract. These results suggest that PP extract has the potential to reduce insulin resistance in GK rats.

Abnormal insulin secretion, rather than insulin resistance, is a major cause for the development of diabetes in GK rats (Kimura et al., 1982). Improvement of insulin sensitivity in GK rats has little effect on alleviation of hyperglycemic state. However, the major causes of insulin resistance in KK‐A^y^ mice are excessive eating as well as a genetic background of the development in obesity‐induced diabetes (Iwatsuka, Shino, & Suzuoki, 1970). Owing to these reasons, they are suitable experimental animals for determining the effects of PP extract on insulin resistance of obesity‐induced type 2 diabetes mellitus. To investigate the effect of PP extract on obesity‐induced diabetes, we used KK‐A^y^ mice fed a diet containing PP extract in the present study.

## 2 MATERIALS AND METHODS

### 2.1 Preparation of PP extract

The PP extract was prepared as described previously (Izuchi et al., 2009). In brief, dried PP powder was extracted with ethanol; then, a methyl *tert*‐butyl ether‐soluble fraction of the extract was collected and evaporated. Chemical components present in PP extract are shown in Supporting Information Table S1.

### 2.2 Experimental animals and diets

Five‐week‐old male KK‐A^y^/TaJcl mice were purchased from CREA Japan (Tokyo, Japan). They were housed individually in plastic cages and maintained at a temperature of 22 ± 1°C, under a 12‐hr light/dark cycle (lights on from 08:00 to 20:00 daily). They were fed a commercial diet (AIN‐93G; Oriental Yeast, Tokyo, Japan) for a week and then divided into two groups with similar average body weight: a control diet group (CD, *n* = 7) fed a commercial diet and a PP extract diet group (PD, *n* = 6) fed a commercial diet containing 1 g/kg of PP extract. The mice were allowed free access to food and drinking water.

After 8 weeks, each mouse was fasted for 3 hr and then anesthetized intraperitoneally with pentobarbital. Blood samples were collected from the carotid artery, treated with heparin, and centrifuged at 800×*g*. The supernatants were collected and stored at −20°C until use. Livers were excised and placed in RNA*later* ^®^ (Ambion^®^, Austin, TX, USA) for subsequent RNA analysis. The protocol for the animal experiments was approved by the Animal Use Committee of the Faculty of Agriculture at The University of Tokyo (P10‐403).

### 2.3 Measurement of plasma biochemical parameters

Aspartate aminotransferase, alanine aminotransferase, total cholesterol, triacylglycerol, nonesterified fatty acid, LDL‐ and HDL‐cholesterol, glucose, total ketone bodies, and glycoalbumin levels in plasma were analyzed using 7180 clinical analyzer (Hitachi High‐Technologies, Tokyo, Japan). Commercial ELISA kits were used to measure the concentrations of plasma adiponectin (Otsuka Pharmaceutical Co. Ltd, Tokyo, Japan), insulin, leptin (Morinaga Institute of Biological Science Inc., Yokohama, Japan).

### 2.4 Separation and quantitative determination of nonesterified fatty acids

The lipid extracts from livers were mixed with an appropriate amount of undecanoic acid as an internal standard and then separated by silica gel TLC. A part of the nonesterified fatty acids were methyl‐esterified with boron trifluoride–methanol complex (14% in methanol). The reaction solutions were mixed with hexane and saturated saline. The hexane layers were filtered (pore size 0.45 μm, ADVANTEC, Tokyo, Japan) and analyzed by GC‐TOF MS. Deal conditions are shown in Supporting Information.

The concentration of each fatty acid in the lipid extract was determined by comparing the values of peak areas in total ion chromatogram with those in methyl‐esterified fatty acid standards: palmitic, stearic, oleic, and linoleic acids.

### 2.5 DNA microarray experiments and data analysis

The livers of four mice from each group were subjected to DNA microarray analysis based on the levels of plasma total ketone bodies close to the average value. Total RNA was isolated from the liver using TRIzol^®^ (Life Technologies, Carlsbad, CA, USA) reagent and purified using an RNeasy mini kit (QIAGEN, Hilden, Germany). The quality and quantity of the purified total RNA were checked by agarose gel electrophoresis and spectrophotometry, respectively. DNA microarray was performed using Affymetrix GeneChip^®^ system. Details are described in Supporting Information.

Gene‐annotation enrichment analysis of the differentially expressed genes (DEGs, a false discovery rate <0.1) was performed using the Database for Annotation, Visualization and Integrated Discovery (Retrieved from http://david.abcc.ncifcrf.gov/) (da Huang, Sherman, & Lempicki, 2009) and QuickGO (Retrieved from http://www.ebi.ac.uk/QuickGO/) (Binns et al., 2009). *p*‐Values from modified Fisher's exact test (Hosack, Dennis, Sherman, Lane, & Lempicki, 2003) were used to extract statistically overrepresented Gene Ontology (GO) terms from DEGs. GO terms with *p*‐values less than 0.05 were considered to be significantly enriched. Additionally, upstream factor analysis of DEGs was performed using Ingenuity Pathway Analysis (IPA, QIAGEN) software.

### 2.6 Statistical analysis

Each value was expressed as mean ± SEM. Differences between the groups were calculated using an unpaired Student's *t* test and a *p *<* *0.05 was considered statistically significant.

## 3 RESULTS

### 3.1 Effects of PP extract administration on plasma biochemical parameters and hepatic fatty acids

We evaluated the effects of PP extract administration on plasma biochemical parameters and nonesterified fatty acids of KK‐A^y^ mice (Table 1). Plasma total ketone bodies were significantly lower in PD than in CD mice (*p = *0.014). The detected fatty acids mostly belonged to four types: palmitic acid (C16:0), stearic acid (C18:0), oleic acid (C18:1 [n‐9]), and linoleic acid (C18:2 [n‐6]). Liver palmitic acid content was significantly higher in PD than in CD mice (*p *=* *0.027). The other fatty acid levels tended to be higher in PD than in CD mice.

**Table 1:** Plasma biochemical parameters and hepatic nonesterified fatty acid levels in KK‐A^y^ mice administered with PP extract for 8 weeks

	CD	PD
Plasma		
Aspartate aminotransferase (IU/L)	145 ± 16.5	125 ± 13.8
Alanine aminotransferase (IU/L)	37 ± 3.2	34 ± 2.1
Total cholesterol (mg/dl)	141 ± 3.9	138 ± 7.8
Triacylglycerol (mg/dl)	237 ± 22.7	193 ± 32.1
Nonesterified fatty acid (μEq/L)	508 ± 47.8	459 ± 43.4
LDL‐cholesterol (mg/dl)	7 ± 0.4	6 ± 0.5
HDL‐cholesterol (mg/dl)	80 ± 1.6	80 ± 3.7
Glucose (mg/dl)	604 ± 45.9	596 ± 27.0
Total ketone bodies (μmol/L)	94 ± 8.6^a^	68 ± 4.0
Glycoalbumin (%)	9.6 ± 0.77	10.0 ± 0.53
Insulin (ng/ml)	58.3 ± 45.3	30.9 ± 7.6
Adiponectin (μg/ml)	10.2 ± 0.0	10.2 ± 0.0
Leptin (ng/ml)	57.5 ± 3.1	49.3 ± 3.4
Hepatic nonesterified fatty acid		
Palmitic acid (mg/g)	0.327 ± 0.017	0.482 ± 0.057^a^
Stearic acid (mg/g)	0.082 ± 0.008	0.105 ± 0.012
Oleic acid (mg/g)	0.131 ± 0.007	0.223 ± 0.048
Linoleic acid (mg/g)	0.104 ± 0.009	0.165 ± 0.036

a Student's *t* test, *p *<* *0.05 vs CD. Values are represented as means ± SEM (*n* = 6).

### 3.2 Alterations of hepatic gene expression profile by PP extract and identification of enriched GO terms in DEGs

**Figure 1:**
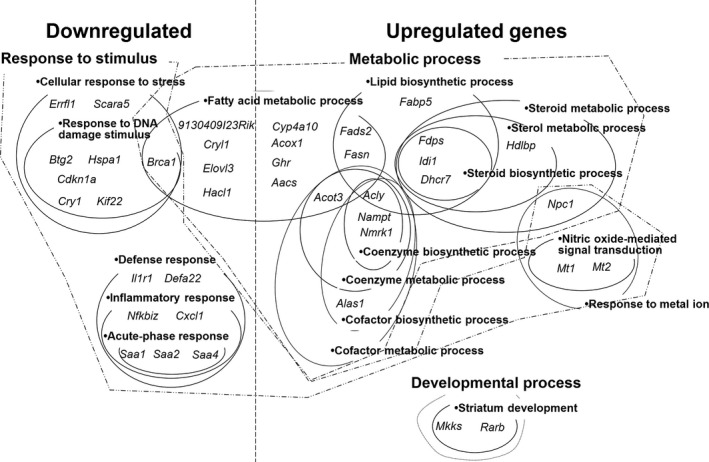
Venn and Euler diagrams showing the relationship between significantly enriched Gene Ontology (GO) terms in differentially expressed genes (DEGs) following administration of persimmon peel extract in KK‐A^y^ mice. The DEGs included in the GO terms are indicated as gene symbols. Left side of the dashed line shows downregulated genes and the right side shows upregulated genes. “Metabolic process” (dot‐dashed line), “response to stimulus” (dot‐dot‐dashed line), and “development process” (dotted line) show the common ancestors of the enriched GO terms surrounded by the respective lines

It has been suggested that a decrease in total ketone body levels and increase in hepatic palmitic acid of PD mice are caused by an alteration of hepatic fatty acid metabolism. Accordingly, we evaluated the effect of PP extract administration on hepatic lipid metabolism using a DNA microarray. DEGs (Supporting Information Table S3 and S4) were classified into functional GO categories. GO terms that were significantly enriched within the upregulated and downregulated sets of DEGs are summarized (Supporting Information Tables S5 and S6) and DEGs contained in each GO term are shown in Figure 1.

Most of the overrepresented GO terms in the upregulated genes were classified into “metabolic process.” The “metabolic process” cluster included three terms associated with lipids: “fatty acid metabolic process,” “lipid biosynthetic process,” and “steroid metabolic process.”

The terms associated with lipids included fatty acid synthase (*Fasn*), acetoacetyl‐CoA synthetase (*Aacs*), cytochrome P450 [family 4, subfamily a, polypeptide 10] (*Cyp4a10*), acyl‐CoA thioesterase 3 (*Acot3*), fatty acid‐binding protein 5 (*Fabp5*), fatty acid desaturase 2 (*Fads2*), acyl‐CoA oxidase 1 (*Acox1*), farnesyl diphosphate synthetase (*Fdps*), isopentenyl diphosphate delta isomerase (*Idi1*), and 7‐dehydrocholesterol reductase (*Dhcr7*).

Most of the overrepresented GO terms in the downregulated genes were classified into “response to stimulus.” “Response to stimulus” included two clusters: “cellular response to stress” and “defense response.” “Cellular response to stress” included cyclin‐dependent kinase inhibitor (*Cdkn1a*), B‐cell translocation gene 2 (*Btg2*), kinesine family member 22 (*Kif22*), breast cancer 1 (*Brca1*), and heat shock protein 1A (*Hspa1*). “Defense response” included interleukin 1 receptor, type 1 (*Il1r1*), chemokine (C‐X‐C motif) ligand 1 (*Cxcl1*), and SAA subtype 1, 2, and 4 (*Saa 1, 2,* and *4*).

**Figure 2:**
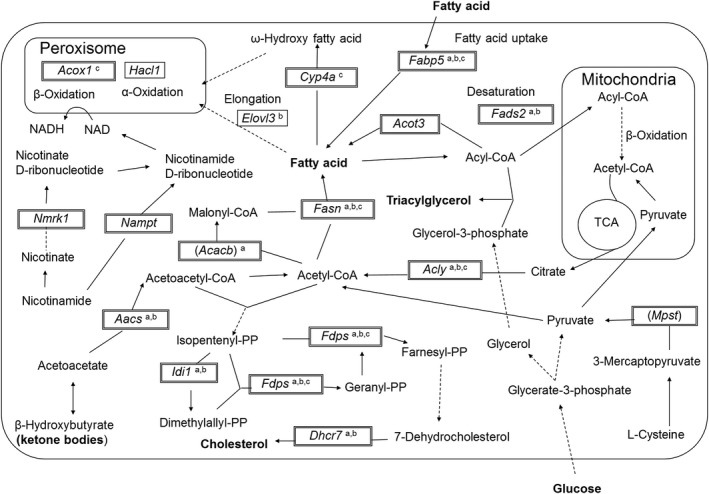
Relationship between metabolic pathways regulated by the products of DEGs and their metabolites. Upregulated factors are surrounded by double lines and downregulated factors by single lines. Factors in parentheses are not represented in the GO terms included in (Figure 1). ^a^These factors are regulated by the sterol regulatory element‐binding protein 1. ^b^These factors are regulated by the sterol regulatory element‐binding protein 2. ^c^These factors are regulated by the peroxisome proliferator‐activated receptor gamma (PPARγ)

Upstream factors regulating the DEGs were predicted using IPA. The predicted transcription factors that activated DEGs upstream after PP extract administration were SREBP (sterol regulatory element‐binding protein) ‐1 and ‐2 and PPARγ (peroxisome proliferator‐activated receptor gamma). Within the upregulated genes by PP extract administration, the genes regulated by SREBP‐1 and ‐2 and PPARγ were as follows: *Fasn*,* Aacs*,* Fabp5*,* Acly*,* Fads2*,* Acacb*,* Fdps*,* Idi1*,* Dhcr7*,* Acox1*,* Cyp4a11*, and *Cyp4a14* (Figure 2 and Table 2).

**Table 2:** Predicted upstream regulators of DEGs by PP extract administration

Upstream transcription factors	*z*‐Score	Regulated genes by PP extract	
		Predicted upregulation genes	Predicted downregulation genes
Predicted activation factor			
SREBP‐2	2.552	***Aacs*** a *, **Acly**,* *Cdkn1a* b *, **Dhcr7**,** Fabp5**,** Fads2**,** Fasn**,** Fdps**,** Idi1**,** Thrsp***	
PPARγ	2.251	***Acly, Acox1,*** *Brca1* *,* *Cdkn1a* *, **Cidea, Cidec, Cyp4a11**,** Cyp4a14**,** Fabp5**,** Fasn**,** Fdps**,* *Gdf15* *,* *Igfbp1* *,* *Plin5* *, **Rarb**,* *Saa1* *,* *Socs3* *, **Vnn1***	*Tsc22d3* *,* *Cxcl2*
SREBP‐1	2.123	***Aacs**,** Acacb**,** Acly**,* *Cdkn1a* *, **Dhcr7**,** Fabp5**,** Fads2**,** Fasn**,** Fdps**,** Idi1**,** Npc1**,* *Nr0b2* *, **Thrsp***	
Predicted inhibition factor			
NR3C1	−2.476	*Adck3* *, **Bmf**,* *Brca1* *,* *Btg1* *,* *Cdkn1a* *, **Cidea**,** Cidec**,* *Cxcl2* *,* *Egr1* *,* *Errfi1* *,* *Fos* *, **Gadd45a**,* *Igfbp1* *,* *Mfsd2a* *, **Mt1**,** Nampt**,* *Nfil3* *,* *Nr0b2* *, **Rtn4**,* *Tat* *,* *Tsc22d3*	
STAT3	−2.382	***Bmf**,* *Cdkn1a* *, **Col3a1**,* *Cxcl2* *,* *Egr1* *,* *Fo* *s, **Gadd45a**,* *Igfbp1* *,* *Il1r1* *,* *Il6r* *, **Mt1**,** Nampt**,* *Nfkbiz* *,* *Saa1* *,* *Serpinb1* *,* *Serpine2* *,* *Socs3*	***Fasn**,*
CREB1	−2.214	*Alas1* *,* *Btg2* *,* *Cdkn1a* *,* *Cxcl2* *,* *Egr1* *,* *Errfi1* *, **Fam73a**,** Fasn**,* *Fos* *, **Gadd45a**,** Idi1**,* *Lcn2* *,* *Mfsd2a* *,* *Nfil3* *, **Nrep**,* *Tfrc*	
NF‐κB	−2.091	*Cdkn1a* *,* *Cxcl2* *, **Dbp**,* *Egr1* *, **Fabp5**,* *Fos* *,* *Gdf15* *, **Igfbp2**,* *Iglv1* *,* *Lcn2* *, **Nampt**,* *Nfkbiz* *, **Rgs16**,* *Saa1* *,* *Socs3* *,* *Tfrc*	***Gadd45a**,*

Note. ^a^Bold letters indicate upregulated genes and ^b^underlined letters are downregulated genes by PP extract.

On the other hand, the predicted transcription factors that inhibited DEGs upstream after PP extract administration were NR3C1 (nuclear receptor subfamily 3, group C, member 1), STAT3 (signal transducer and activator of transcription 3), CREB1 (cAMP responsive element binding protein 1), NF‐κB (nuclear factor‐kappa B) (Table 2).

## 4 DISCUSSION

### 4.1 Effects of PP extract administration on energy metabolism

We used KK‐A^y^ mice to evaluate the effects of PP extract administration on obesity‐induced diabetes and observed that the total ketone body levels in the plasma of KK‐A^y^ mice were decreased by the administration of PP extract. In diabetes mellitus, reduction of glucose uptake into the cells owing to deficiency of insulin signal enhances β‐oxidation of fatty acids and results in further generation of ketone bodies as a byproduct. In contrast, hepatic nonesterified palmitic acid levels in KK‐A^y^ mice were significantly increased by the administration of PP extract. It is suggested that fatty acid synthesis is promoted by PP extract because most of the de novo synthesized fatty acid is palmitic acid (Kuhajda et al., 1994).

In addition to the increase in nonesterified palmitic acid in the liver, detailed pathway analysis suggested the enhancement of fatty acid biosynthesis in PD mice, such as upregulation of *Fasn* and *Acacb* (Figure 2), genes encoding the key enzymes for fatty acid synthesis (Wakil, Stoops, & Joshi, 1983). Moreover, a decrease in plasma total ketone bodies can be explained by the upregulation of *Aacs*. *Aacs*, encoding the ketone body‐utilizing enzyme AACS (Endemann, Goetz, Edmond, & Brunengraber, 1982), which converts acetoacetate derived from ketone bodies to acetoacetyl‐CoA and then promotes a reaction in the direction of acetyl‐CoA production.

The activation of hepatic PPARγ and SREBP‐1 and 2 by PD‐administered mice was predicted by IPA. SREBP‐1c is an important factor in controlling blood glucose and regulating the expression of glycolysis‐associated genes and fatty acid biosynthesis (Shimano et al., 1997). We infer that SREBP‐1c activated in the liver of PD mice upregulated the expression of *Fasn* and *Acc* and fatty acid synthesis.

Peroxisome proliferator‐activated receptor gamma is a nuclear receptor associated with adipocyte differentiation and fat accumulation. Therefore, activation of PPARγ is a possible way to improve insulin resistance and hyperglycemia. Activation of PPARγ induces the upregulation of genes associated with lipogenesis (Way et al., 2001), and decreases the formation of ketone bodies (Fujiwara, Yoshioka, Yoshioka, Ushiyama, & Horikoshi, 1988). PPARγ activated by PP extract can also promote fatty acid synthesis. However, it was presumed that the PPARγ activity of PP extract was low, and did not contribute to the improvement of insulin resistance and hyperglycemia.

### 4.2 Effects of PP extract administration on inflammatory responses

The present study revealed that the expression of genes associated with inflammation such as *Il1r1*,* Il6ra*, and *Saa 1*,* 2*, and *4* was downregulated in the livers of PD mice (Supporting Information Table S4). Inflammatory cytokines, including IL‐1 and IL‐6, are released from macrophages and adipocytes and lead to the pathogenesis of insulin resistance and development of type 2 diabetes mellitus (Hotamisligil, 2006). These cytokines induce the expression of acute‐phase inflammatory markers, C‐reactive protein (CRP), and SAA in the liver (Steel & Whitehead, 1994).

Inhibition of STAT3 and NF‐κB signals, which are upstream activators of *Il6 and Il1* (Libermann & Baltimore, 1990), was predicted by IPA. Obese diabetic KK‐A^y^ mice display obesity‐induced chronic inflammation (Kato et al., 2001). We assumed that the reduction in reactive oxygen species (ROS) caused by the administration of PP extract containing antioxidants (carotenoids, polyphenols, vitamin E, etc.) would suppress inflammation caused by the overproduction of ROS in diabetes mellitus (Roman‐Pintos, Villegas‐Rivera, Rodriguez‐Carrizalez, Miranda‐Diaz, & Cardona‐Munoz, 2016). Furthermore, downregulation of genes involved in the “cellular response to stress” (*Cdkn1*,* Btg2*,* Kif22*,* Brca1*,* Hspa1*,* Cry1*,* Kif22*,* Errfl1*,* Scara5*) is likely due to the reduction of stressors, such as ROS, by PP extract.

Our results suggest that the suppression of inflammatory cytokines can be attributed to a reduction in oxidative stress caused by antioxidants in the extract, and subsequent downregulation of downstream STAT3 and NF‐κB pathways. Suppressing inflammation through the intake of PP extract may lead to a decreased risk of diabetes development or progression.

### 4.3 Effect of PP extract on animal models of diabetes mellitus

In our previous study, the GK rats administered with PP extract indicated enhanced tyrosine phosphorylation of insulin receptor β‐subunit and upregulation of *Srebp‐1c* gene expression in the liver at mRNA level (Izuchi et al., 2011). These results suggested that the upregulation of *Srebp‐1c* gene expression in the liver of PP extract‐administered GK rats was induced by the enhancement of insulin receptor signaling, and consequently the expression of the genes involved in fatty acid synthesis, *Fasn*,* Acaca*, and *Acacb*, was upregulated. These results were consistent with the hepatic gene expression profile of the extract‐administered KK‐Ay mice. The functional characteristics of hepatic DEGs in GK rats administered PP extract showed upregulation of the genes related to glycolysis and fatty acid synthesis, and downregulation of the genes related to β‐oxidation and gluconeogenesis. We expect that the effects of the extract on hepatic gene expression are induced by PPARγ activation, because the alterations were similar to the hepatic gene expression profile displayed in the presence of a PPARγ agonist (Way et al., 2001). This hepatic gene expression is probably caused by quercetin and ursolic acid present in PP extract, which are potential PPARγ agonists (Wang et al., 2014; Way et al., 2001).

Moreover, our previous study showed that GK rats administered with the extract reduced plasma ALT levels and downregulated the expression of hepatic *Il1r* and *Crp* genes with inflammation. In the present study, KK‐Ay mice administered PP extract also showed a gene expression pattern similar to that of the GK rats. Fat‐soluble antioxidants such as β‐cryptoxanthin, quercetin, and vitamin E in the extract could act as anti‐inflammatory agents and contribute to the improvement of insulin resistance.

In conclusion, we propose that PP extract promotes insulin receptor activity through multiple mechanisms, including the activation of PPARγ and anti‐inflammatory effects, through which it alters fatty acid synthesis through SREBP‐1c and PPARγ. PP extract administration altered hepatic gene expression in both obese and nonobese diabetic animals, but its effect was limited due to its very low activity, which may not overcome abnormal insulin secretion and excessive eating. Therefore, the extract could contribute to support improvement in insulin resistance alongside diet restriction and medication use.

## CONFLICT OF INTEREST

No potential conflict of interest was reported by the authors.

## ETHICAL STATEMENTS

These animal experiments were reviewed and approved by the Animal Use Committee of the Faculty of Agriculture at The University of Tokyo (Japan).
