# Supplementary material for: Hepatic fatty acid biosynthesis in KK‐Ay mice is modulated by administration of persimmon peel extract: A DNA microarray study
Source: Food Sci Nutr. 2018 Jul 20;6(6):1657–63. doi: 10.1002/fsn3.728 (PMC6145309; doi:10.1002/fsn3.728)
Supplement: Supplementary file 1 [file FSN3-6-1657-s001.docx]

| **Table S1 Compounds contained in PP extract** | |
| --- | --- |
| **Compounds** | **Content (mg/g)** |
| **Fatty acids** |  |
| Myristic acid (14:0) | 13.4 |
| Palmitic acid (16:0) | 49.6 |
| Palmitoleic acid (16:1) | 21.6 |
| Stearic acid (18:0) | 4.6 |
| Oleic acid (18:1 n-9) | 2.6 |
| *cis*-Vaccenic acid (18:1 n-7) | 79.0 |
| Linoleic acid (18:2 n-6) | 7.6 |
| α-Linolenic acid (18:3 n-3) | 77.3 |
| γ-Linolenic acid (18:3 n-6) | 0.3 |
| Arachidic acid (20:0) | 1.9 |
| Heneicosanoic acid (21:0) | 1.2 |
| Behenic acid (22:0) | 4.0 |
| **Triterpenoid** |  |
| Ursolic acid | 112.1 |
| **Vitamin** |  |
| α-Tocopherol | 14.9 |
| **Carotenoids^a^** |  |
| β-Cryptoxanthin | 13.4 |
| Zeaxanthin | 4.0 |
| Lutein | 3.5 |
| β-Carotene | 1.4 |
| Violaxanthin | 0.2 |
| α-Carotene | 0.2 |
| **Polyphenol aglycons^a^** |  |
| Quercetin | 2.6 |
| Kaempferol | 0.5 |
| Gallic acid | 0.4 |
| *p*-Hydroxybenzoic acid | 0.1 |
| Ellagic acid | 0.1 |
| Myricetin | 0.1 |
| ^a^ These values cited our previously published datum^11)^. | |

| **Table S2.** Final body weight and total food intake of KK-A^y^ mice administered with PP extract for 8 weeks | | |
| --- | --- | --- |
|  | CD | PD |
| Final body weight (g) | 43.97 ± 1.39 | 42.53 ± 1.07 |
| Total food intake (g) | 383.56 ± 9.63 | 359.33 ± 9.42 |
| Hepatic triacylglycerol (mg/g) | 66.09 ± 3.73 | 70.98 ± 8.13 |
| Hepatic cholesterol (mg/g) | 3.57 ± 0.13 | 3.38 ± 0.16 |
| Values are represented as mean ± SEM of seven animals in CD and six animals in PD. | | |

**Table S3 List of upregulated probe sets (FDR<0.1) in the liver of KK-A^y^ mice by PP extract.**

| Probe set ID | Gene Title | Gene Symbol | FDR |
| --- | --- | --- | --- |
| 1426037_a_at | regulator of G-protein signaling 16 | Rgs16 | 0 |
| 1455265_a_at | regulator of G-protein signaling 16 | Rgs16 | 0 |
| 1423257_at | cytochrome P450, family 4, subfamily a, polypeptide 14 | Cyp4a14 | 0 |
| 1429203_at | RIKEN cDNA 2410076I21 gene | 2410076I21Rik | 0 |
| 1444009_at | Ras association (RalGDS/AF-6) domain family member 4 | Rassf4 | 0.002 |
| 1424853_s_at | cytochrome P450, family 4, subfamily a, polypeptide 10 /// cytochrome P450, family 4, subfamily a, polypeptide 31 | Cyp4a10 /// Cyp4a31 | 0.001667 |
| 1418486_at | vanin 1 | Vnn1 | 0.002857 |
| 1438617_at | serine (or cysteine) peptidase inhibitor, clade A (alpha-1 antiproteinase, antitrypsin), member 7 | Serpina7 | 0.0025 |
| 1441957_x_at | RIKEN cDNA 2410076I21 gene | 2410076I21Rik | 0.002222 |
| 1429639_at | glycerophosphocholine phosphodiesterase GDE1 homolog (S. cerevisiae) | Gpcpd1 | 0.002 |
| 1417956_at | cell death-inducing DNA fragmentation factor, alpha subunit-like effector A | Cidea | 0.001818 |
| 1423152_at | vesicle-associated membrane protein, associated protein B and C | Vapb | 0.001667 |
| 1459948_at | --- | --- | 0.001538 |
| 1416773_at | WEE 1 homolog 1 (S. pombe) | Wee1 | 0.002143 |
| 1443147_at | --- | --- | 0.002 |
| 1429144_at | glycerophosphocholine phosphodiesterase GDE1 homolog (S. cerevisiae) | Gpcpd1 | 0.003125 |
| 1447845_s_at | vanin 1 | Vnn1 | 0.002941 |
| 1438211_s_at | D site albumin promoter binding protein | Dbp | 0.002778 |
| 1418174_at | D site albumin promoter binding protein | Dbp | 0.003158 |
| 1451452_a_at | regulator of G-protein signaling 16 | Rgs16 | 0.003 |
| 1439622_at | Ras association (RalGDS/AF-6) domain family member 4 | Rassf4 | 0.00381 |
| 1427473_at | glutathione S-transferase, mu 3 | Gstm3 | 0.003636 |
| 1440443_at | RIKEN cDNA E030016H06 gene | E030016H06Rik | 0.003478 |
| 1456081_a_at | acetoacetyl-CoA synthetase | Aacs | 0.00375 |
| 1451190_a_at | SH3-binding kinase 1 | Sbk1 | 0.0044 |
| 1423797_at | acetoacetyl-CoA synthetase | Aacs | 0.005769 |
| 1452417_x_at | immunoglobulin kappa chain variable 28 (V28) /// immunoglobulin kappa constant /// immunoglobulin kappa joining 1 /// immunoglobulin kappa variable 4-53 /// immunoglobulin kappa variable 6-23 /// immunoglobulin kappa chain variable 8-30 | Igk-V28 /// Igkc /// Igkj1 /// Igkv4-53 /// Igkv6-23 /// Igkv8-30 | 0.006296 |
| 1439189_at | folliculin interacting protein 2 | Fnip2 | 0.008571 |
| 1442331_at | --- | --- | 0.008621 |
| 1422925_s_at | acyl-CoA thioesterase 3 | Acot3 | 0.009 |
| 1427660_x_at | immunoglobulin kappa chain variable 28 (V28) /// immunoglobulin kappa constant /// immunoglobulin kappa joining 1 /// immunoglobulin kappa variable 4-53 /// immunoglobulin kappa variable 6-23 /// immunoglobulin kappa chain variable 8-30 | Igk-V28 /// Igkc /// Igkj1 /// Igkv4-53 /// Igkv6-23 /// Igkv8-30 | 0.01 |
| 1418989_at | cathepsin E | Ctse | 0.01125 |
| 1422973_a_at | thyroid hormone responsive SPOT14 homolog (Rattus) | Thrsp | 0.012727 |
| 1449519_at | growth arrest and DNA-damage-inducible 45 alpha | Gadd45a | 0.012647 |
| 1427474_s_at | glutathione S-transferase, mu 3 | Gstm3 | 0.013429 |
| 1421087_at | period homolog 3 (Drosophila) | Per3 | 0.014167 |
| 1431038_at | Ras association (RalGDS/AF-6) domain family member 4 | Rassf4 | 0.016486 |
| 1438050_x_at | predicted gene 9222 | Gm9222 | 0.016316 |
| 1440730_at | --- | --- | 0.016154 |
| 1417126_a_at | ribosomal protein L22 like 1 | Rpl22l1 | 0.016 |
| 1458701_at | glycerophosphocholine phosphodiesterase GDE1 homolog (S. cerevisiae) | Gpcpd1 | 0.019024 |
| 1417168_a_at | ubiquitin specific peptidase 2 | Usp2 | 0.019286 |
| 1422557_s_at | metallothionein 1 | Mt1 | 0.019302 |
| 1420603_s_at | retinoic acid early transcript 1, alpha /// retinoic acid early transcript beta /// retinoic acid early transcript gamma /// retinoic acid early transcript delta /// retinoic acid early transcript 1E | Raet1a /// Raet1b /// Raet1c /// Raet1d /// Raet1e | 0.02 |
| 1458832_at | --- | --- | 0.021778 |
| 1427883_a_at | collagen, type III, alpha 1 | Col3a1 | 0.021739 |
| 1418356_at | mercaptopyruvate sulfurtransferase | Mpst | 0.021915 |
| 1419031_at | fatty acid desaturase 2 | Fads2 | 0.024167 |
| 1428942_at | metallothionein 2 | Mt2 | 0.024082 |
| 1443838_x_at | fatty acid desaturase 2 | Fads2 | 0.0256 |
| 1435207_at | DIX domain containing 1 | Dixdc1 | 0.02549 |
| 1444518_at | Acyl-Coenzyme A oxidase 1, palmitoyl | Acox1 | 0.025962 |
| 1421116_a_at | reticulon 4 | Rtn4 | 0.026981 |
| 1454159_a_at | insulin-like growth factor binding protein 2 | Igfbp2 | 0.028704 |
| 1426294_at | hyaluronan and proteoglycan link protein 1 | Hapln1 | 0.028364 |
| 1416021_a_at | fatty acid binding protein 5, epidermal /// predicted gene 3601 | Fabp5 /// Gm3601 | 0.029643 |
| 1419590_at | cytochrome P450, family 2, subfamily b, polypeptide 9 | Cyp2b9 | 0.029123 |
| 1424737_at | thyroid hormone responsive SPOT14 homolog (Rattus) | Thrsp | 0.029483 |
| 1423804_a_at | predicted gene 13502 /// isopentenyl-diphosphate delta isomerase | Gm13502 /// Idi1 | 0.029153 |
| 1451548_at | uridine phosphorylase 2 | Upp2 | 0.030167 |
| 1423086_at | Niemann Pick type C1 | Npc1 | 0.030164 |
| 1425646_at | nicotinamide riboside kinase 1 | Nmrk1 | 0.033871 |
| 1448048_at | nicotinamide riboside kinase 1 | Nmrk1 | 0.040317 |
| 1415859_at | eukaryotic translation initiation factor 3, subunit C | Eif3c | 0.04 |
| 1438558_x_at | forkhead box Q1 | Foxq1 | 0.041846 |
| 1458176_at | Period homolog 3 (Drosophila) | Per3 | 0.042424 |
| 1436736_x_at | DNA segment, human D4S114 | D0H4S114 | 0.044328 |
| 1423418_at | farnesyl diphosphate synthetase | Fdps | 0.043676 |
| 1424969_s_at | uridine phosphorylase 2 | Upp2 | 0.046812 |
| 1457141_at | --- | --- | 0.052 |
| 1422627_a_at | McKusick-Kaufman syndrome protein | Mkks | 0.051268 |
| 1440134_at | cytochrome P450, family 4, subfamily a, polypeptide 31 | Cyp4a31 | 0.056111 |
| 1451666_at | ATP citrate lyase | Acly | 0.06 |
| 1427052_at | acetyl-Coenzyme A carboxylase beta | Acacb | 0.059324 |
| 1415988_at | high density lipoprotein (HDL) binding protein | Hdlbp | 0.059333 |
| 1429906_at | --- | --- | 0.058684 |
| 1454906_at | retinoic acid receptor, beta | Rarb | 0.058312 |
| 1448619_at | 7-dehydrocholesterol reductase | Dhcr7 | 0.060256 |
| 1445047_at | expressed sequence C79246 | C79246 | 0.066709 |
| 1437002_at | family with sequence similarity 73, member A | Fam73a | 0.073375 |
| 1419806_at | high density lipoprotein (HDL) binding protein | Hdlbp | 0.079383 |
| 1453473_a_at | dynein light chain Tctex-type 1A /// dynein light chain Tctex-type 1D /// dynein light chain Tctex-type 1C /// dynein light chain Tctex-type 1F | Dynlt1a /// Dynlt1b /// Dynlt1c /// Dynlt1f | 0.087317 |
| 1423978_at | SH3-binding kinase 1 | Sbk1 | 0.090723 |
| 1453588_at | carbonic anhydrase 3 | Car3 | 0.091905 |
| 1455320_at | nicotinamide phosphoribosyltransferase | Nampt | 0.092471 |
| 1417169_at | ubiquitin specific peptidase 2 | Usp2 | 0.09314 |
| 1454880_s_at | BCL2 modifying factor | Bmf | 0.092874 |
| 1452260_at | cell death-inducing DFFA-like effector c | Cidec | 0.093182 |
| 1417904_at | DNA cross-link repair 1A, PSO2 homolog (S. cerevisiae) | Dclre1a | 0.099213 |
| 1444032_at | --- | --- | 0.099556 |
| 1423828_at | fatty acid synthase | Fasn | 0.098571 |
| 1455665_at | LON peptidase N-terminal domain and ring finger 1 | Lonrf1 | 0.0975 |

**Table S4 List of downregulated probe sets (FDR<0.1) in the liver of KK-A^y^ mice by PP extract.**

| Probe set ID | Gene Title | Gene Symbol | FDR |
| --- | --- | --- | --- |
| 1421679_a_at | cyclin-dependent kinase inhibitor 1A (P21) | Cdkn1a | 0.01 |
| 1449347_a_at | X-linked lymphocyte-regulated 4A /// X-linked lymphocyte-regulated 4B /// X-linked lymphocyte-regulated 4C /// X-linked lymphocyte-regulated 4D, pseudogene | Xlr4a /// Xlr4b /// Xlr4c /// Xlr4d-ps | 0.005 |
| 1420357_s_at | X-linked lymphocyte-regulated 3A /// X-linked lymphocyte-regulated 3B /// X-linked lymphocyte-regulated 3C | Xlr3a /// Xlr3b /// Xlr3c | 0.006666667 |
| 1424629_at | breast cancer 1 | Brca1 | 0.005 |
| 1416666_at | serine (or cysteine) peptidase inhibitor, clade E, member 2 | Serpine2 | 0.008 |
| 1425295_at | eosinophil-associated, ribonuclease A family, member 11 | Ear11 | 0.006666667 |
| 1450264_a_at | choline kinase alpha | Chka | 0.008571429 |
| 1424638_at | cyclin-dependent kinase inhibitor 1A (P21) | Cdkn1a | 0.01 |
| 1426215_at | dopa decarboxylase | Ddc | 0.008888889 |
| 1423447_at | caseinolytic peptidase X (E.coli) | Clpx | 0.008 |
| 1442025_a_at | --- | --- | 0.009090909 |
| 1428669_at | brain expressed myelocytomatosis oncogene | Bmyc | 0.01 |
| 1419209_at | chemokine (C-X-C motif) ligand 1 | Cxcl1 | 0.009230769 |
| 1450788_at | serum amyloid A 1 | Saa1 | 0.009285714 |
| 1419874_x_at | zinc finger and BTB domain containing 16 | Zbtb16 | 0.01 |
| 1449326_x_at | serum amyloid A 2 | Saa2 | 0.010625 |
| 1418746_at | paroxysmal nonkinesiogenic dyskinesia | Pnkd | 0.01 |
| 1419075_s_at | serum amyloid A 1 | Saa1 | 0.012222222 |
| 1433634_at | interferon regulatory factor 2 binding protein 2 | Irf2bp2 | 0.012631579 |
| 1433691_at | protein phosphatase 1, regulatory (inhibitor) subunit 3C | Ppp1r3c | 0.0135 |
| 1441326_at | ceruloplasmin | Cp | 0.013333333 |
| 1457644_s_at | chemokine (C-X-C motif) ligand 1 | Cxcl1 | 0.014090909 |
| 1439357_at | interleukin 17 receptor E | Il17re | 0.013478261 |
| 1437019_at | family with sequence similarity 25, member C | Fam25c | 0.014166667 |
| 1419319_at | serum amyloid A 4 | Saa4 | 0.0144 |
| 1427422_at | predicted gene 6484 | Gm6484 | 0.015384615 |
| 1417065_at | early growth response 1 | Egr1 | 0.015555556 |
| 1425281_a_at | TSC22 domain family, member 3 | Tsc22d3 | 0.015 |
| 1441855_x_at | chemokine (C-X-C motif) ligand 1 | Cxcl1 | 0.016551724 |
| 1434280_at | --- | --- | 0.016333333 |
| 1420772_a_at | TSC22 domain family, member 3 | Tsc22d3 | 0.017096774 |
| 1418932_at | nuclear factor, interleukin 3, regulated | Nfil3 | 0.0171875 |
| 1418949_at | growth differentiation factor 15 | Gdf15 | 0.019090909 |
| 1424022_at | oxidative stress induced growth inhibitor 1 | Osgin1 | 0.021764706 |
| 1431283_at | --- | --- | 0.023142857 |
| 1451557_at | tyrosine aminotransferase | Tat | 0.0275 |
| 1457534_at | Predicted gene, 19710 | Gm19710 | 0.031351351 |
| 1424029_at | TSPY-like 4 | Tspyl4 | 0.031315789 |
| 1433668_at | proline-rich nuclear receptor coactivator 1 | Pnrc1 | 0.031025641 |
| 1449854_at | nuclear receptor subfamily 0, group B, member 2 | Nr0b2 | 0.033 |
| 1431679_at | RIKEN cDNA 2510042H12 gene | 2510042H12Rik | 0.032682927 |
| 1425557_x_at | TSC22 domain family, member 3 | Tsc22d3 | 0.037142857 |
| 1426851_a_at | nephroblastoma overexpressed gene | Nov | 0.036511628 |
| 1423813_at | kinesin family member 22 | Kif22 | 0.036590909 |
| 1452951_at | RIKEN cDNA 2410089E03 gene | 2410089E03Rik | 0.036 |
| 1421091_at | serine (or cysteine) peptidase inhibitor, clade A (alpha-1 antiproteinase, antitrypsin), member 12 | Serpina12 | 0.035869565 |
| 1433733_a_at | cryptochrome 1 (photolyase-like) | Cry1 | 0.038297872 |
| 1418918_at | insulin-like growth factor binding protein 1 | Igfbp1 | 0.0375 |
| 1445695_at | --- | --- | 0.037755102 |
| 1457605_at | --- | --- | 0.0424 |
| 1456388_at | ATPase, class VI, type 11A | Atp11a | 0.04254902 |
| 1436857_at | defensin, alpha, 21 /// defensin, alpha, 22 | Defa21 /// Defa22 | 0.041923077 |
| 1434067_at | expressed sequence AI662270 | AI662270 | 0.045471698 |
| 1443059_at | hydroxysteroid (17-beta) dehydrogenase 11 | Hsd17b11 | 0.046666667 |
| 1435319_at | inositol hexaphosphate kinase 2 | Ip6k2 | 0.048727273 |
| 1417066_at | aarF domain containing kinase 3 | Adck3 | 0.047857143 |
| 1422070_at | alcohol dehydrogenase 4 (class II), pi polypeptide | Adh4 | 0.047017544 |
| 1433898_at | --- | --- | 0.046896552 |
| 1430983_at | protein disulfide isomerase associated 6 | Pdia6 | 0.046101695 |
| 1421041_s_at | predicted gene 3776 /// glutathione S-transferase, alpha 1 (Ya) /// glutathione S-transferase, alpha 2 (Yc2) | Gm3776 /// Gsta1 /// Gsta2 | 0.046333333 |
| 1456960_at | --- | --- | 0.047540984 |
| 1433181_at | RIKEN cDNA 4930431N21 gene | 4930431N21Rik | 0.048225806 |
| 1433974_at | selenophosphate synthetase 1 | Sephs1 | 0.048412698 |
| 1420722_at | elongation of very long chain fatty acids (FEN1/Elo2, SUR4/Elo3, yeast)-like 3 | Elovl3 | 0.04796875 |
| 1448950_at | interleukin 1 receptor, type I | Il1r1 | 0.047538462 |
| 1443422_at | RIKEN cDNA 2410089E03 gene | 2410089E03Rik | 0.046818182 |
| 1430689_at | RIKEN cDNA 2810019C22 gene | 2810019C22Rik | 0.048059701 |
| 1445378_at | --- | --- | 0.047794118 |
| 1423100_at | FBJ osteosarcoma oncogene | Fos | 0.047391304 |
| 1436224_at | kinesin family member 1C | Kif1c | 0.047571429 |
| 1456219_at | zinc finger protein of the cerebellum 5 | Zic5 | 0.048591549 |
| 1428112_at | mesencephalic astrocyte-derived neurotrophic factor | Manf | 0.049444444 |
| 1421040_a_at | glutathione S-transferase, alpha 2 (Yc2) | Gsta2 | 0.049863014 |
| 1451382_at | ChaC, cation transport regulator 1 | Chac1 | 0.049864865 |
| 1453582_at | choline kinase alpha | Chka | 0.049733333 |
| 1416250_at | B cell translocation gene 2, anti-proliferative | Btg2 | 0.049605263 |
| 1435021_at | gamma-aminobutyric acid (GABA) A receptor, subunit beta 3 | Gabrb3 | 0.053506494 |
| 1439163_at | zinc finger and BTB domain containing 16 | Zbtb16 | 0.054487179 |
| 1436590_at | protein phosphatase 1, regulatory (inhibitor) subunit 3B | Ppp1r3b | 0.057088608 |
| 1455282_x_at | aminolevulinic acid synthase 1 | Alas1 | 0.05875 |
| 1419520_at | N-acetyltransferase 8 (GCN5-related, putative) | Nat8 | 0.05962963 |
| 1455234_at | UDP-Gal:betaGlcNAc beta 1,3-galactosyltransferase, polypeptide 1 | B3galt1 | 0.060609756 |
| 1427747_a_at | lipocalin 2 | Lcn2 | 0.062048193 |
| 1452661_at | transferrin receptor | Tfrc | 0.062261905 |
| 1428223_at | major facilitator superfamily domain containing 2A | Mfsd2a | 0.067058824 |
| 1453498_x_at | STEAP family member 3 | Steap3 | 0.066627907 |
| 1417311_at | cysteine rich protein 2 | Crip2 | 0.068275862 |
| 1451204_at | scavenger receptor class A, member 5 (putative) | Scara5 | 0.068409091 |
| 1445054_at | --- | --- | 0.067640449 |
| 1449047_at | 2-hydroxyacyl-CoA lyase 1 | Hacl1 | 0.069777778 |
| 1441915_s_at | perilipin 5 | Plin5 | 0.069010989 |
| 1431182_at | heat shock protein 8 | Hspa8 | 0.071521739 |
| 1427572_at | --- | --- | 0.070967742 |
| 1424126_at | aminolevulinic acid synthase 1 | Alas1 | 0.070744681 |
| 1416318_at | serine (or cysteine) peptidase inhibitor, clade B, member 1a | Serpinb1a | 0.08 |
| 1446551_at | Predicted gene, 16794 | Gm16794 | 0.080104167 |
| 1459557_at | --- | --- | 0.079896907 |
| 1451313_a_at | RIKEN cDNA 1110067D22 gene | 1110067D22Rik | 0.080612245 |
| 1452445_at | solute carrier family 41, member 2 | Slc41a2 | 0.082121212 |
| 1434068_s_at | expressed sequence AI662270 | AI662270 | 0.0826 |
| 1427820_at | --- | --- | 0.082574257 |
| 1430523_s_at | immunoglobulin lambda variable 1 | Iglv1 | 0.084901961 |
| 1422257_s_at | cytochrome P450, family 2, subfamily b, polypeptide 10 | Cyp2b10 | 0.085436893 |
| 1452416_at | interleukin 6 receptor, alpha | Il6ra | 0.085192308 |
| 1437918_at | RIKEN cDNA 4930539E08 gene | 4930539E08Rik | 0.086952381 |
| 1421954_at | v-crk sarcoma virus CT10 oncogene homolog (avian)-like | Crkl | 0.08754717 |
| 1427127_x_at | heat shock protein 1B | Hspa1b | 0.08728972 |
| 1416795_at | crystallin, lambda 1 | Cryl1 | 0.086851852 |
| 1426083_a_at | B cell translocation gene 1, anti-proliferative | Btg1 | 0.086605505 |
| 1457403_at | RIKEN cDNA 9130409I23 gene | 9130409I23Rik | 0.087545455 |
| 1433816_at | mitochondrial carrier triple repeat 1 | Mcart1 | 0.088198198 |
| 1456767_at | leucine rich repeat and fibronectin type III domain containing 3 | Lrfn3 | 0.088660714 |
| 1425837_a_at | CCR4 carbon catabolite repression 4-like (S. cerevisiae) | Ccrn4l | 0.089823009 |
| 1456212_x_at | suppressor of cytokine signaling 3 | Socs3 | 0.090964912 |
| 1455899_x_at | suppressor of cytokine signaling 3 | Socs3 | 0.091391304 |
| 1436392_s_at | transcription factor AP-2, gamma | Tfap2c | 0.092155172 |
| 1419816_s_at | ERBB receptor feedback inhibitor 1 | Errfi1 | 0.093162393 |
| 1441717_at | DNA segment, Chr 9, ERATO Doi 596, expressed | D9Ertd596e | 0.097033898 |
| 1425645_s_at | cytochrome P450, family 2, subfamily b, polypeptide 10 | Cyp2b10 | 0.096218487 |
| 1421656_at | sprouty homolog 2 (Drosophila) | Spry2 | 0.096083333 |
| 1435536_at | DNA-damage inducible protein 2 | Ddi2 | 0.096115702 |
| 1452730_at | ribosomal protein S4, Y-linked 2 | Rps4y2 | 0.09557377 |
| 1417483_at | nuclear factor of kappa light polypeptide gene enhancer in B cells inhibitor, zeta | Nfkbiz | 0.094796748 |
| 1447700_x_at | synovial sarcoma translocation gene on chromosome 18-like 1 | Ss18l1 | 0.096048387 |
| 1419318_at | serum amyloid A 4 | Saa4 | 0.09744 |
| 1437250_at | melanoregulin | Mreg | 0.096746032 |
| 1450272_at | tumor necrosis factor (ligand) superfamily, member 8 | Tnfsf8 | 0.096929134 |

| **Table S5.** Significantly enriched GO terms in the upregulated gene set | | | |
| --- | --- | --- | --- |
| GO-ID | GO terms | Number of genes | EASE scores  (*p*-values from modified Fisher’s exact test) |
| ***Metabolic process***^a)^ | |  |  |
| GO:0006631 | Fatty acid metabolic process | 7 | 0.0001 |
| GO:0008610 | Lipid biosynthetic process | 7 | 0.0015 |
| GO:0008202 | Steroid metabolic process | 5 | 0.0059 |
| GO:0006694 | Steroid biosynthetic process | 3 | 0.0406 |
| GO:0016125 | Sterol metabolic process | 5 | 0.0050 |
| GO:0016126 | Sterol biosynthetic process | 3 | 0.0082 |
| GO:0008203 | Cholesterol metabolic process | 4 | 0.0038 |
| GO:0006695 | Cholesterol biosynthetic process | 3 | 0.0049 |
| GO:0051186 | Cofactor metabolic process | 5 | 0.0086 |
| GO:0051188 | Cofactor biosynthetic process | 4 | 0.0080 |
| GO:0006732 | Coenzyme metabolic process | 4 | 0.0251 |
| GO:0009108 | Coenzyme biosynthetic process | 3 | 0.0345 |
| ***Response to stimulus***^a)^ | |  |  |
| GO:0010038 | Response to metal ion | 3 | 0.0218 |
| GO:0046688 | Response to copper ion | 2 | 0.0316 |
| GO:0010273 | Detoxification of copper ion | 2 | 0.0137 |
| GO:0007263 | Nitric oxide mediated signal transduction | 2 | 0.0091 |
| GO:0055069 | Zinc ion homeostasis | 2 | 0.0360 |
| GO:0006882 | Cellular zinc ion homeostasis | 2 | 0.0316 |
| ***Developmental process***^a)^ | |  |  |
| GO:0021756 | Striatum development | 2 | 0.0448 |
| ^a)^GO terms indicated in italic and bold styles are not significant but are common ancestors of GO terms listed below them. | | | |

| **Table S6.** Significantly enriched GO terms in the downregulated gene set | | | |
| --- | --- | --- | --- |
| GO-ID | GO terms | Number of genes | EASE scores  (*p*-values from modified Fisher’s exact test) |
| ***Metabolic process***^a)^ | |  |  |
| GO:0006631 | Fatty acid metabolic process | 5 | 0.0239 |
| ***Response to stimulus*** ^a)^ | |  |  |
| GO:0033554 | Cellular response to stress | 8 | 0.0119 |
| GO:0006974 | Response to DNA damage stimulus | 6 | 0.0320 |
| GO:0006952 | Defense response | 7 | 0.0314 |
| GO:0006954 | Inflammatory response | 5 | 0.0426 |
| GO:0006953 | Acute-phase response | 3 | 0.0153 |
| ^a)^GO terms indicated in italic and bold styles are not significant but are common ancestors of GO terms listed below them. | | | |

**Supplemental material and methods**

**1 Preparing lipid extracts from livers**

The liver tissue samples were frozen in liquid nitrogen and stored at −80°C until use. Livers were homogenized with a disposable tube and pestle; the samples were placed in a filter paper thimble and lipids were extracted with diethyl ether by using SER148 Soxhlet extractors (VELP scientifica, Usmate Velate, Italy). The lipid extracts were dried before dissolution and dilution to 10 mL with 2-propanol.

**2 Measurement of triacylglycerol levels in liver**

Triacylglycerol content of livers was measured by the acetylacetone method [1]. We used 1 M acetic acid containing 0.5% sodium metaperiodate as an oxidizing reagent and an aqueous solution containing 0.25% (v/v) acetylacetone, 13.35% (v/v) 2-propanol, 0.67 M ammonium acetate, and 0.53 M acetate as a color reagent. The lipid solution was hydrolyzed by adding 5% aqueous potassium hydroxide at 50°C for 15 min. The hydrolyzed solution was mixed with oxidizing and coloring reagents and heated at 50°C for 40 min. After cooling, the concentration of triacylglycerol in the solution was determined using a standard calibration curve of tripalmitin plotted at an absorbance of 410 nm with µQuant microplate spectrophotometer (BioTek, Winooski, VT, USA).

**3 Measurement of cholesterol levels in liver**

Cholesterol levels were measured using previously reported methods[2].We used an acetic anhydride/acetate/sulfuric acid = 20:10:1 (v/v/v) mixture containing 2% sodium sulfate, as Liebermann–Burchard (LB) reagent. The lipid solutions were dried and then dissolved in 2% ethanolic potassium hydroxide. After heating at 40°C for 55 min, the solution was mixed with petroleum ether and water. To the dried petroleum ether layer, LB reagent was added, followed by incubation at 25°C for 30 min. After cooling on ice, the concentration of cholesterol in the solution was determined using a standard cholesterol calibration curve plotted at an absorbance of 620 nm with microplate spectrophotometer.

**4 Extraction of non-esterified fatty acids from hepatic lipid**

The lipid extracts from livers were mixed with an appropriate amount of undecanoic acid as an internal standard and then separated by silica gel TLC with a developing solvent mixture of hexane/diethyl ether/acetic acid = 80:30:1 (v/v/v). The separated lipids were stained with iodine vapor. A part of the non-esterified fatty acids was scraped from the developed plates into a glass tube with a screw cap. The non-esterified fatty acids was were methyl esterified with boron trifluoride–methanol complex (14% in methanol) at 100°C for 7 min. The reaction solutions were mixed with hexane and saturated saline. The hexane layers were filtered (pore size 0.45 µm, ADVANTEC, Tokyo, Japan).

**5 Measurement condition of GC-TOFMS**

Analysis was performed using an Agilent 7890A gas chromatograph (Agilent Technologies, Santa Clara, CA, USA) coupled with a JMS-T100GCV AccuTOF mass spectrometer (JEOL, Tokyo, Japan). The system was equipped with a SP-2380 capillary column (100 m × 0.25 mm id, 0.2 µm film thickness, Sigma-Aldrich, St. Louis, MO, USA). The sample (1 µL) was injected with an autosampler at a split ratio of 20:1. The injector temperature was 250°C, and the carrier gas (helium) flow rate through the column was 1 mL/min. The column temperature was held at 50°C for 2 min, then raised at a rate of 20°C /min from 50 to 140°C and 4°C /min from 140 to 240°C, and held at 240°C for 8.5 min. The ion source temperature, voltage, and current were 250°C, 70 V, and 300 µA, respectively. Mass spectra were collected from m/z 35 to 650.

**5 DNA microarray experiment and data analysis**

DNA microarray analysis was performed as described previously [3]. Briefly, biotinylated aRNA was obtained from 100 ng of purified total RNA using GeneChip^®^ 3′ IVT Express Kit (Affymetrix, Santa Clara, CA, USA). The aRNA was purified, fragmented, and hybridized to GeneChip^®^ Mouse Genome 430 2.0 array containing over 45,000 probe sets representing well-characterized mouse genes. Following hybridization at 45°C for 16 h, the array was washed and labeled with phycoerythrin. Fluorescence signals were scanned using the GeneChip^®^ system. GeneChip^®^ Command Console software was used to convert the array images to the signal intensity of each probe (CEL files). The data discussed in this publication have been deposited in NCBI's Gene Expression Omnibus[4] and are accessible through GEO Series accession number GSE67573 (http://www.ncbi.nlm.nih.gov/geo/query/acc.cgi?acc=GSE67573).

The CEL files were quantified with a model-based expression index (MBEI) [5] using the statistical language R [6] and Bioconductor [7]. Hierarchical clustering was performed using the ‘pvclust()’ function in R [8]. To identify differentially expressed genes (DEGs), the rank product method [9] was applied to the quantified data, with the number of permutations set at 1,000. Probe sets with a false discovery rate < 0.1 were considered as having different expression levels (being differently expressed) between two groups. The annotation file for the array was downloaded from the Affymetrix website (November 2, 2012, Mouse430_2.na33.annot.csv).

Gene-annotation enrichment analysis of the differentially expressed genes (DEGs, a false discovery rate < 0.1) was performed using the Database for Annotation, Visualization and Integrated Discovery and QuickGO. Expression Analysis Systematic Explorer scores, which are *p*-values from modified Fisher’s exact test [13], were used to extract statistically overrepresented Gene Ontology (GO) terms from DEGs.

**Reference for supporting information**

[1] Fletcher, M. J., A colorimetric method for estimating serum triglycerides. *Clinica Chimica Acta* 1968, *22*, 393-397.

[2] Huang, T. C., Chen, C. P., Wefler, V., Raftery, A., A stable reagent for the Liebermann-Burchard reaction. Application to rapid serum cholesterol determination. *Analytical Chemistry* 1961, *33*, 1405-1407.

[3] Suyama, T., Okada, S., Ishijima, T., Iida, K.*, et al.*, High phosphorus diet-induced changes in NaPi-IIb phosphate transporter expression in the rat kidney: DNA microarray analysis. *PLOS ONE* 2012, *7*, e29483.

[4] Edgar, R., Domrachev, M., Lash, A. E., Gene Expression Omnibus: NCBI gene expression and hybridization array data repository. *Nucleic Acids Res* 2002, *30*, 207-210.

[5] Li, C., Wong, W. H., Model-based analysis of oligonucleotide arrays: expression index computation and outlier detection. *Proc. Natl. Acad. Sci. U. S. A.* 2001, *98*, 31-36.

[6] The R Development Core Team, *R: A Language and Environment for Statistical Computing.*, R Foundation for Statistical Computing, Vienna, Austria 2008.

[7] Gentleman, R. C., Carey, V. J., Bates, D. M., Bolstad, B.*, et al.*, Bioconductor: open software development for computational biology and bioinformatics. *Genome Biol.* 2004, *5*, R80.

[8] Suzuki, R., Shimodaira, H., Pvclust: an R package for assessing the uncertainty in hierarchical clustering. *Bioinformatics* 2006, *22*, 1540-1542.

[9] Breitling, R., Armengaud, P., Amtmann, A., Herzyk, P., Rank products: a simple, yet powerful, new method to detect differentially regulated genes in replicated microarray experiments. *FEBS Lett.* 2004, *573*, 83-92.
